# Ketamine’s effect on inflammation and kynurenine pathway in depression: A systematic review

**DOI:** 10.1177/02698811211026426

**Published:** 2021-06-26

**Authors:** Emma Kopra, Valeria Mondelli, Carmine Pariante, Naghmeh Nikkheslat

**Affiliations:** 1 Department of Psychological Medicine, Institute of Psychiatry, Psychology and Neuroscience, King’s College London, UK; 2 National Institute for Health Research (NIHR) Mental Health Biomedical Research Centre at South London and Maudsley NHS Foundation Trust and King’s College London, UK

**Keywords:** Ketamine, inflammation, cytokine, kynurenine, depression

## Abstract

**Background:**

Ketamine is a novel rapid-acting antidepressant with high efficacy in treatment-resistant patients. Its exact therapeutic mechanisms of action are unclear; however, in recent years its anti-inflammatory properties and subsequent downstream effects on tryptophan (TRP) metabolism have sparked research interest.

**Aim:**

This systematic review examined the effect of ketamine on inflammatory markers and TRP–kynurenine (KYN) pathway metabolites in patients with unipolar and bipolar depression and in animal models of depression.

**Methods:**

MEDLINE, Embase, and PsycINFO databases were searched on October 2020 (1806 to 2020).

**Results:**

Out of 807 initial results, nine human studies and 22 animal studies on rodents met the inclusion criteria. Rodent studies provided strong support for ketamine-induced decreases in pro-inflammatory cytokines, namely in interleukin (IL)-1β, IL-6, and tumor necrosis factor (TNF)-α and indicated anti-inflammatory effects on TRP metabolism, including decreases in the enzyme indoleamine 2,3-dioxygenase (IDO). Clinical evidence was less robust with high heterogeneity between sample characteristics, but most experiments demonstrated decreases in peripheral inflammation including in IL-1β, IL-6, and TNF-α. Preliminary support was also found for reduced activation of the neurotoxic arm of the KYN pathway.

**Conclusion:**

Ketamine appears to induce anti-inflammatory effects in at least a proportion of depressed patients. Suggestions for future research include investigation of markers in the central nervous system and examination of clinical relevance of inflammatory changes.

## Introduction

Major depressive disorder (MDD) is among the leading causes of disability worldwide, contributing to high human and societal costs (Ferrari et al., 2013). For decades, depression has primarily been treated with antidepressants targeting monoamine function; however, about one-third of patients are considered treatment-resistant to such medications, lacking adequate response to two or more antidepressant trials (Nemeroff, 2007). For those who respond, effects take usually 2–4 weeks to be noticeable and a large proportion eventually relapse (Gaynes et al., 2009). In bipolar disorder (BD), where depressive episodes alternate with manic episodes and antidepressant response rates are even lower (Pacchiarotti et al., 2013) with fewer available treatment options than in MDD (Vieta et al., 2010). Novel treatments and drug targets are therefore urgently needed.

Ketamine, a noncompetitive antagonist of the glutamate N-methyl-D-aspartate (NMDA) receptor, has been referred to as one of the most important discoveries in antidepressant research in half a century (Duman and Aghajanian, 2012). It has been used as a dissociative anesthetic since the 1960s, but its therapeutic potential was not discovered until the year 2000 (Berman et al., 2000). Since then, numerous studies have replicated ketamine’s antidepressant and anti-suicidal effects in both unipolar and bipolar depression (Coyle and Laws, 2015; Wilkinson et al., 2018) with high response rates of up to 60%–70% in treatment-resistant patients (Bobo et al., 2016; Diazgranados et al., 2010; Murrough et al., 2013a). Ketamine is fast acting with effects emerging within hours (Bobo et al., 2016) making it a beneficial tool, especially for patients in acute risk of self-harm and suicide (Larkin and Beautrais, 2011). Ketamine’s effects peak approximately 1-day post-infusion and last around 1 week (Corriger and Pickering, 2019; Kishimoto et al., 2016) but can be prolonged with repeated infusions (Murrough et al., 2013b; Zheng et al., 2018). The (S)-isomer of ketamine (esketamine) was approved as a medication for treatment-resistant MDD in nasal spray form in the United States in 2019 (FDA, 2019).

The exact mechanisms behind ketamine’s antidepressant effects are yet to be fully understood. However, its NMDA receptor antagonist property has been postulated to play a central role, the therapeutic mechanisms are believed to be much more complex. This is supported by the existence of several other NMDA receptor antagonists without antidepressant properties (Newport et al., 2015), as well as preclinical research on the pharmacology and efficacy of ketamine’s enantiomers (Hashimoto, 2019; Jelen et al., 2020; Yang et al., 2015a) and metabolites (Hashimoto, 2019; Zanos et al., 2016). In recent years, there has been an increasing interest in ketamine’s anti-inflammatory properties. Ketamine has been shown to attenuate heightened inflammation in animal models, in human blood in vitro (De Kock et al., 2013; Loix et al., 2011), and in surgical contexts when used as an anesthetic or antihyperalgesic (Dale et al., 2012; Loix et al., 2011).

The anti-inflammatory effects of ketamine have been of particular interest given that the association between inflammation and depression is well-established. This relationship is likely to be bidirectional, with high inflammation levels (Khandaker et al., 2014; Valkanova et al., 2013) and presence of inflammation-related diseases (Benros et al., 2013; Sforzini et al., 2019; Wang et al., 2018) increasing not only the risk of developing MDD and BD, but also vice versa (Andersson et al., 2015; Rosenblat and McIntyre, 2017). Increased inflammatory markers, specifically C-reactive protein (CRP), interleukin (IL)-1, IL-6, and tumor-necrosis factor alpha (TNF-α), have been observed in the blood and cerebrospinal fluid (CSF) as well as in postmortem brain samples of MDD and BD patients (Enache et al., 2019; Osimo et al., 2020; Raison et al., 2006; Rosenblat et al., 2014). Interestingly, inflammation is prevalent, especially in treatment-resistant depression (Cattaneo et al., 2020; Strawbridge et al., 2015; Yang et al., 2019); and longitudinal investigations have found patients with higher baseline inflammation to be less likely to respond to traditional antidepressants (Cattaneo et al., 2013, 2016; Strawbridge et al., 2015).

Among the main mechanisms, inflammation is suggested to induce depressive symptoms through its effects on the tryptophan (TRP)–kynurenine (KYN) pathway, as supported by studies in which activation of KYN pathway and increased depressive symptoms were observed in patients with hepatitis C virus infection undergoing interferon (IFN)-α treatment (Raison et al., 2010). Indeed, pro-inflammatory cytokines IFN-γ, IFN-α, IL-1, and TNF-α activate the enzyme indoleamine 2,3-dioxygenase (IDO), which metabolizes TRP into KYN instead of serotonin (Maes et al., 2007). The pro-inflammatory cytokine IL-1β further activates the enzyme 3-monooxygenase (KMO; Zunszain et al., 2012), which converts KYN into its neurotoxic microglial by-products, such as 3-hydroxykynurenine (3-HK), 3-hydroxyanthranilic acid (3-HAA) and eventually quinolinic acid (QUIN; Ogyu et al., 2018; Parrott et al., 2016). These metabolites contribute to neurotoxicity through numerous mechanisms and can also further exacerbate inflammation. For instance, QUIN agonizes the NMDA receptor inducing glutamate excitotoxicity and contributing to a signaling cascade that leads to reduced brain-derived neurotropic factor (BDNF), protein synthesis, and synaptogenesis (Lugo-Huitrón et al., 2013), whereas 3-HK increases reactive oxygen species in the brain, contributing to oxidative stress and neuronal apoptosis, especially in the hippocampus (Colín-González et al., 2013). Under normal conditions, KYN is mainly metabolized in astrocytes by kynurenine aminotransferases (KATs) to kynurenic acid (KynA). KynA is an antagonist of the NMDA receptor and to a lesser extent the α7-nicotinic acetylcholine and AMPA receptors, through which it exerts neuroprotective and anti-inflammatory effects, enhances synaptic plasticity, and clears excess glutamate in the brain (Ganong and Cotman, 1986; Potter et al., 2010).

Evidence is rapidly growing on the role of inflammation in association with KYN pathway abnormalities and dysregulation of KYN metabolites in patients who have committed suicide (Bryleva and Brundin, 2017). However, the data is less robust for the whole MDD population. A recent systematic review observed decreased KYN and KynA in depressed patients, alongside increased QUIN in antidepressant-free patients, compared to healthy controls (Ogyu et al., 2018); however, some studies have found no differences between KYN pathway metabolites of depressed patients and healthy controls in peripheral (Hughes et al., 2012) nor central samples (Hestad et al., 2017; Miller et al., 2008). Decreased TRP among patients with no changes in KYN or its downstream components has been also observed (Gabbay et al., 2010; Hughes et al., 2012), suggesting potential involvement of alternative mechanisms in the depletion of TRP.

Understanding ketamine’s potential effect on inflammatory proteins and TRP metabolism would increase our understanding of the biological mechanisms behind its antidepressant effect and help identify pharmacological targets for other antidepressants. Crucially, understanding ketamine’s immunomodulatory mechanisms could inform us of potential predictors of response and therefore help reduce unnecessary treatment trials. Changes in cytokines have previously been found to be associated with antidepressant response to traditional antidepressants (Cattaneo et al., 2013; Lanquillon et al., 2000), and a recent systematic review concluded that treatment-resistant patients with higher inflammation have better response to medication with anti-inflammatory characteristics including ketamine (Yang et al., 2019). Identification of predictors of response as well as development of novel pharmacotherapies are of particular interest given concerns surrounding ketamine’s side-effects and potential risks of long-term use (Short et al., 2018).

Thus far research on the effect of ketamine on inflammation has yielded mixed reports with heterogeneity in methodologies and samples complicating evidence synthesis. The aim of this systematic review is to analyze current evidence on the effect of ketamine on inflammatory markers and TRP–KYN pathway metabolites in unipolar and bipolar depression, as well as in preclinical studies employing animal models of depression.

## Methods

### Search strategy

Electronic literature databases MEDLINE ([R] and Epub Ahead of Print, In-Process, In-Data-Review & Other Non-Indexed Citations and Daily; 1946 to October 4, 2020), APA PsycInfo (1806 to September Week 4, 2020), and Embase (1974 to 2020 Week 40) were searched through Ovid interface (https://ovidsp.ovid.com/) to find relevant studies. The following combination of keywords was entered: Ketamine AND Depress* AND Inflammat*/cytokine*/interleukin/c-reactive protein/CRP/tumor necrosis factor*/TNF*/interferon/IFN*/kynuren*/KYNA/quinolinic/QUIN/QA. Results were limited to publications in English language. No restrictions were placed regarding publication year. Reference sections of included studies and review articles on the topic were screened to identify additional relevant publications. The final search was performed on October 5th, 2020.

### Selection of literature

References were imported into the RefWorks citation manager tool (ProQuest, Ann Arbor, MI, USA) for screening of results. Participants in human studies were required to have a current Diagnostic and Statistical Manual of Mental Disorders (DSM) or International Classification of Diseases (ICD) diagnosis of either MDD or BD and to be experiencing a depressive episode at the time of the study. Animal studies were required to use established animal models of depression such as lipopolysaccharide- (LPS) or stress-induced depression.

Studies were required to include in vivo administration of ketamine. Studies were excluded if ketamine was administered as an adjunct therapy with another pharmacological or psychological treatment, though participants were allowed to be maintained on their current medications. Human studies were required to measure biomarkers at baseline and at least once posttreatment and provide a within-subject analysis of the change, or at minimum provide sufficient data for interpretation of this change. Animal studies were required to include comparison of posttreatment biomarkers between ketamine group and a control group with otherwise identical treatment but without administration of an active drug.

### Quality assessment

The quality of human studies was evaluated with the following parameters: (1) blinding of patients and investigators, (2) absence of adjunct medication, (3) number of outcome measurement points, (4) appropriate statistical analyses, and (5) completeness of data and reporting. Animal studies’ quality was assessed based on (1) depression model, (2) clarity of protocol and similar treatment of groups, (3) appropriate statistical analyses; and (4) completeness of data and reporting. Each parameter was awarded a maximum score of two, depending on how well each study fulfilled the criterion (0 = low/absent, 1 = partial, and 2 = full). A total quality score was generated by summing the scores for all parameters, with a maximum score of 10 for human studies and 8 for animal studies.

## Results

### Study selection

The initial search yielded 848 results, reducing to 807 after limiting results to English language and 581 after deduplication. About 62 articles were selected for full-text assessment after the initial title and abstract screening, of which 31 were included in the qualitative analysis. Nine of the included studies were on humans and 22 on rodents. A flow diagram of the search process with reasons for exclusions is presented in Supplemental Figure S1.

### Description of selected studies

#### Human studies

A summary of human study characteristics is shown in Table 1. All studies were published between 2015 and 2020. Seven studies measured inflammatory markers, the most widely measured being IL-6 (*n* = 7) and TNF-α (*n* = 6); and five studies measured TRP–KYN metabolites, the most commonly measured being KYN (*n* = 5) and TRP (*n* = 4). All studies measured protein levels of the markers. The samples of studies by Kadriu et al. (2019) and Park et al. (2017) overlapped, and therefore for the former we will only report results for KYN metabolites but not for cytokines, which are analyzed more thoroughly and in a larger sample by Park et al. (2017).

**Table 1.** Study characteristics and results of human studies.

Study	Sample	Medication	Design	Intervention	Outcome measures	Findings
Allen et al. (2018)	17 TRD	15/17 (88%) Medicated	Open label, repeated measures	0.5 mg/kg i.v. ketamine once a week for 3 weeks (=3 infusions)	Whole blood IL-6, IL-8, IL-10, IFN-γ; and KYN, KynA, TRP, KYN/TRP ratio, KynA/KYN ratio; at baseline, 2 h and 1-week post each infusion, and 24 h post-first infusion	No significant changes after ketamine
Ireland	47% Female patients					In responders (HDRS reduction >50%; *n* = 13), trend towards decreased KYN 2 h after first infusion (*p* = 0.067), and reduced KYN/TRP ratio at 24 h after first infusion (*p* = 0.054)
Chen et al. (2018)	71 TRD (*n* = 23–24/group)	Medications continued (no numbers given)	Double-blind RCT	Single 0.5 mg/kg i.v. ketamine; 0.2 mg/kg i.v. ketamine; or i.v. saline	Serum CRP, IL-6, and TNF-α at baseline, 40 min, 240 min, day 3, and day 7 post-infusion	IL-6 and TNF-α levels differed significantly over time in all groups (*p* = 0.002, *p* = 0.001). TNF-α was significantly lower at 40 and 240 min post-infusion than at baseline for the 0.5 mg/kg group (*p* < 0.05)
Taiwan	75% Female patients					
Kadriu et al. (2019) ^a^	39 TRBD	All patients on mood stabilizers only	Sample drawn from a randomized placebo-controlled crossover study	Single 0.5 mg/kg i.v. ketamine over 40 min	Plasma IDO, KYN, KynA, and QA at baseline, 230 min, day 1 and day 3 post-infusion.	IDO levels significantly lower than baseline at all three time points, *t*(34) = −3.05, *p* = 0.004; *t*(34) = −4.12, *p* = 0.0002; *t*(34) = −3.91, *p* = 0.0004 for 230 min, 1 day, and 3 days, respectively.
USA	59% Female patients					KYN significantly increased at 1 day (*t* = 3.69, *p* = 0.0008) and 3 day (*t* = 2.44, *p* = 0.02). KynA significantly increased at 1 day (*t* = 2.85, *p* = 0.007) and 3 day (*t* = 3.36, *p* = 0.002). KYN/KynA ratio significantly increased (*t* = 2.91, *p* = 0.006) and QA/KYN ratio significantly decreased (*t* = −3.31, *p* = 0.002) at day 1.
Kiraly et al. (2017)	33 TRD	Free of medication for ⩾2 weeks	Open label, repeated measures	Single 0.5 mg/kg i.v. ketamine over 40 min	Serum levels of 41 cytokines, chemokines and growth factors at baseline; and 4 h and 24 h post-infusion	Significant decreases in IL-6 (*t* = 2.369, *p* < 0.05), IL-1α (*p* < 0.05, *t* = 2.149), IL-13 (*p* = 0.038), G-CSF (*p* = 0.038), and IP-10/CXCL10 (*p* < 0.0001) at 4 h; alongside decreases in IL-8 (*p* < 0.0001) and PDGF-AA (*p* = 0.024); and increases in IL-7 (*p* < 0.0001) at 24 h
USA						
Moaddel et al. (2018)	29 TRD^b^	Free of medication for ⩾2 weeks	Double-blind, placebo-controlled crossover study	Single 0.5 mg/kg i.v. ketamine or saline over 40 mins, 2 weeks apart	Plasma KYN and TRP at baseline, 40 min (end of infusion), 80 min, 110 min, 230 min, and at days 1, 2 and 3 post-infusion	No significant changes after ketamine
USA						When compared to placebo, changes in KYN/TRP ratio (*p* = 0.013) and KYN (*p* = 0.039), namely a slight initial increase in ketamine group compared to a sharp drop in placebo group
Park et al. (2017) ^a^	49 TRD and 31 TRBD	TRD free of medication for ⩾2 weeks; BD on mood stabilizers only	Sample drawn from double-blind placebo-controlled studies	Single 0.5 mg/kg i.v. ketamine over 40 mins	Plasma IL-2, IL-5, IL-6, IL-8, IL-10, IFN-γ, TNF-α and sTNFR1 at baseline and at 230 min, 1 day and 3 days post-infusion	Significantly increased IL-6 (*F*_3,209_ = 25.51, *p* < 0.001) and decreased sTNFR1 (*F*_3,206_ = 4.27, *p* = 0.006) 230 min post-infusion
USA	51% Female patients					
Yang et al. (2015b)	16 TRD^c^	Free of medication for ⩾2 weeks	Open label, repeated measures	Single 0.5 mg/kg i.v. ketamine over 40 mins	Serum IL-1β, IL-6, and TNF-α; and KYN and TRP; at baseline, 230 mins and 1- and 3-day post-infusion	In the responder group (MADRS reduction >50%; *n* = 12), IL-1β showed significant decrease at 230 mins and 1-day post-infusion (*F* = 4.495, df = 2.602, *p* = 0.013), and IL-6 significant decrease at 230 mins to 3 days post-infusion (*F* = 9.450, df = 2.914, *p* < 0.001)
China/Japan						
Zhan et al. (2020)	60 MDD (TR or suicidal)	All medicated	Open label, repeated measures	Six 0.5 mg/kg i.v. ketamine infusions (40 mins) over a 12-day period	Plasma levels of 19 cytokines at baseline, 24 h after first infusion, and 24 h and 14 days after sixth infusion	Levels of 14 cytokines significantly decreased at 14 days post-sixth infusion; IL-1β, IL-4, IL-5, IL-12p70, IL-23, and GM-CSF (*p* < 0.05); and IL-2, IL-6, IL-7, IL-10, IL-17A, IFN-γ, TNF-α, and fractalkine (*p* < 0.01)
China	63% Female patients					
Zhou et al. (2018)	68 MDD and 16 BD (TR = 73; suicidal = 48)	All medicated	Open label, repeated measures	Six 0.5 mg/kg i.v. ketamine infusions (40 min) over a 12-day period	Serum TRP, KYN, and KynA at baseline, 24 h after first infusion, and 24 h and 14 days after sixth infusion	In responders (MADRS reduction >50%; *n* = 50), KynA and KynA/KYN ratio increased at 24 h after first infusion (KynA: *t* = 2.842, *p* = 0.031; KynA/KYN ratio: *t* = 2.842, *p* = 0.031) and 24 h after sixth infusion (KynA: *t* = −2.946, *p* = 0.023; KynA/KYN ratio: *t* = −2.919, *p* = 0.025)
China	54% Female patients					

3-HK: 3-hydroxykynurenine; 3-HAA: 3-hydroxyanthranilic acid; BD: bipolar disorder; CRP: C-reactive protein; CSF: cerebrospinal fluid; G-CSF: granulocyte colony-stimulating factor; GM-CSF: granulocyte-macrophage colony-stimulating factor; HDRS: Hamilton depression rating scale; IDO: indoleamine 2,3-dioxygenase; IL: interleukin; IFN-α: interferon-alpha; i.v.: intravenous; KYN: kynurenine; KynA: kynurenic acid ; MDD: major depressive disorder; MADRS: Montgomery–Åsberg depression rating scale; TRBD: treatment-resistant bipolar depression; TNF-α: tumor necrosis factor-alpha; TRD: treatment-resistant major depression; TRP: tryptophan.

a Kadriu et al. (2019) and Park et al. (2017) had partially overlapping sample; therefore, cytokine data only reported for Park et al (2017).

b TR criteria required only one failed previous antidepressant trial.

c TR criteria not specified.

The number of patients receiving ketamine ranged from 16 to 84, the combined sample of all included studies totaling 429. Six studies included MDD patients, one BD patients, and two studies included both. Most studies employed treatment-resistant criteria requiring patients to have had at least two previous failed antidepressant trials, except from one study only requiring a single failed trial (Moaddel et al., 2018). Two studies allowed inclusion of nontreatment-resistant patients if these were suicidal (Zhan et al., 2020; Zhou et al., 2018). In four out of six studies, MDD patients were medication free, and in two out of three studies, BD patients were allowed mood stabilizers only. Six studies administered ketamine once, and three had multiple infusions. All studies used a 0.5 mg/kg intravenous dose of ketamine with one study also including a 0.2 mg/kg condition (Chen et al., 2018). Studies included between 2 and 7 post-infusion measurements, from immediately after administration to up to 2-week post-infusion.

Study quality scoring is shown in Supplemental Table S1. Total scores ranged between four and eight out of ten. Statistical analyses were appropriate by most part; however, a few studies did exploratory analyses with several markers without adjusting for multiple comparisons where this would have been considered appropriate. Moaddel et al. (2018) did not conduct a simple within-subject analysis of ketamine-induced changes but rather contrasted effects to a placebo group; therefore, absolute changes in levels had to be interpreted from a graph. Two studies provided only stratified data according to antidepressant responder status.

#### Animal studies

Animal study characteristics are summarized in Table 2. All studies were published between 2013 and 2020. Sixteen studies examined inflammatory markers, one study TRP– KYN pathway metabolites, and five studies measured both. The most measured markers were IL-1β and IL-6, measured in 19 and 18 studies, respectively. All studies measured protein levels of the markers, except from three studies measuring mRNA levels of cytokines and one study measuring mRNA levels of IDO. Twelve studies measured markers in either the brain (most commonly hippocampus or prefrontal cortex) or CSF, seven in blood, and three studies reported levels for both. In 13 studies, tissue or blood samples were taken less than 24 h from treatment, in seven studies 24 h or more, and two did both with different groups of rodents. Ketamine was delivered intraperitoneally with doses ranging from 5 to 20 mg/kg, except from one study with a 100 mg/kg dose (Zhu et al., 2015) and one with an additional 90 mg/kg condition besides a lower-dose group (Verdonk et al., 2019). About 17 studies administered a single ketamine infusion, three injected multiple infusions, and two included both conditions.

**Table 2.** Study characteristics and results of animal studies.

Study	Animals^a^	Model	Intervention	Outcome measures^b^	Findings
Abelaira et al. (2017)	8–10 Adult male Wistar rats	FST	Ketamine 15 mg/kg i.p. or saline	Serum IL-1β, IL-10, and TNF-α	TNF-α increased in ketamine group (*p* < 0.05)
				0.5 h	
Aricioglu et al. (2020)	30 Adult male Wistar albino rats	CUMS for 6 weeks	Ketamine 10 mg/kg i.p. single dose, or daily for 3 weeks (in the last 3 weeks of CUMS), or saline	PFC IL-1β and IL-6 (mRNA)	Both acute and chronic ketamine decreased IL-1β; only acute ketamine decreased IL-6 (*p* < 0.05)
					7 days
Chang et al. (2019)	20 Adult male Wistar rats	LPS	Ketamine 10 mg/kg, i.p. or saline	Hippocampal tissue IL-1β, IL-6, and TNF-α	IL-1β, TNF-α (*p* < 0.01), and IL-6 (*p* < 0.05) significantly lower in ketamine group
					24 h
Clarke et al. (2017)	16 Adult male CD1 mice	LPS (after ketamine)	Ketamine 5 or 10 mg/kg i.p. or saline	Plasma GM-CSF, IFN-γ, IL-10, IL-1β, IL-6, and TNF-α	Both ketamine doses decreased TNF-α (*p* < 0.01). High-dose ketamine decreased IL-1β (*p* < 0.05)
				2h	
Eskelund et al. (2017)	54 FSL rats	FSL; genetic rat depression model	(A) Ketamine 15 mg/kg i.p. or saline; (B) Ketamine 15 mg/kg i.p. or saline every third day for 14 days	Brain and plasma KYN, KynA, QUIN, 3-HK, TRP, and 5-HT	No differences
				4 h	
Ji et al. (2019)	~51 Male Sprague Dawley rats	LPS	Ketamine 6 mg/kg i.p. or saline	Plasma TNF-α, IL-1β, and IL-6.	No differences
				11.5 h	
Li et al. (2019)	Male C57BL/6 mice	LPS	Ketamine 10 mg/kg i.p. or no treatment	Hippocampal IL-1β	Ketamine decreased IL-1β (*p* < 0.001)
				~3 h	
Reus et al. (2017)	8–10 Adult male Wistar rats	FST or Open field + splash test (all after ketamine)	Ketamine 5 mg/kg i.p. or saline	Serum IL-1β, IL-10, and IL-6	No differences
				~1.5 h	
Reus et al. (2015)	10–12 Juvenile male Wistar rats	Maternal deprivation (+ FST on day 13 and 14)	Ketamine 15 mg/kg i.p. or saline, once a day for 14 days	Serum and CSF IL-1β, IL-6, and TNF-α	Ketamine decreased levels of all cytokines; IL-6 in both serum and CSF, IL-1β and TNF-α in serum only (*p* < 0.05)
				~24 h	
Tan et al. (2017)	12 Kunming mice	Chronic restraint stress 4 h/day on 21 days	Ketamine 20 mg/kg i.p. or saline on days 22, 24, and 25	Serum IL-1β, IL-6, and TNF-α	Ketamine decreased levels of all cytokines (*p* < 0.05)
				1 h after last infusion	
Unal et al. (2015)	12–14 Sprague Dawley rats	4 h restraint stress on 7 days	Ketamine 10 mg/kg i.p. or no treatment on day 7	Hippocampal and PFC TNF-α, IL-1β, and IL-6 (mRNA)	In hippocampus, ketamine decreased levels of all cytokines (*p* < 0.05)
				4.5 h	
Verdonk et al. (2019)	18 Adult male knock-in CX3CR1GFP/+ Mice	LPS	Ketamine 10 mg/kg, 90 mg/kg, or placebo	Brain tissue levels of multiple markers incl. IL-1α, IL-1β, IL-6, IL-10, IFN-γ, TNF-α; and TRP, KYN, KynA, QUIN, 5-HT, and 3-HK	Both ketamine doses decreased IL-1α, IL-6, and G-CSF (*p* < 0.01 for 90 mg/kg, *p* < 0.05 for 10 mg/kg)
				32 h	Both ketamine doses decreased QUIN (*p* < 0.01). 90 mg/kg ketamine reduced 3-HK (*p* < 0.0001); and increased KynA (*p* < 0.001), data for 10 mg/kg not given
Walker et al. (2015)	Male albino Wistar rats	ACTH or saline for 14 days (TR rat model)	Ketamine 10 mg/kg i.p. or saline	Plasma IL-6, TNF-α, and CRP.	TR ketamine responders had higher CRP than TR placebo group (*p* < 0.05) and non-TR ketamine group (*p* < 0.001). TR ketamine non-responders had lower TNF-α than TR placebo group (*p* < 0.05)
				~2 h	
Walker et al. (2013)	Juvenile CD-1 and adult C57BL/6J male mice	LPS (after ketamine)	Ketamine 6 mg/kg i.p. or saline	Plasma IL-1β; plasma and brain IL-6, KYN, and TRP; and brain IDO mRNA	No differences
				6 h and 28 h	
Wang et al. (2015)	40 Adult male Wistar rats.	CUMS over 21 days	Ketamine 10 mg/kg i.p. (0.5, 1, 2, or 4 h before behavioral tests) or saline (0.5 h before behavioral tests)	Hippocampal IL-1β, IL-6, TNF-α, IDO, KYN, and TRP	Ketamine decreased IL-1β at 0.5 and 1 h, TNF-α at 2 and 4 h, and IL-6 at all four timepoints (*p* < 0.05).
				1, 1.5, 2.5, or 4.5 h	Ketamine decreased KYN/TRP ratio and IDO at all four timepoints (*p* < 0.05, IDO at 0.5 h *p* < 0.01)
Xie et al. (2017)	30 Male Sprague Dawley rats	Neuropathic pain/SNI	Ketamine 20 mg/kg i.p. or saline	Serum IL-1β, IL-6, TNF-α	In rats with depression-like phenotype (*n* = 12), Ketamine decreased IL-1β and IL-6 (*p* < 0.05). Depression phenotype allocated based on behavioral tests
				3 days	
Yang et al. (2013a)	20 Male Wistar rats	FST	Ketamine 10 mg/kg i.p. or saline	PFC and hippocampal IL-1β and IL-6	Ketamine significantly decreased IL-1β and IL-6 in PFC and hippocampus (*p* < 0.05)
				0.5 h	
Yang et al. (2013b)	20 Male Wistar rats	LPS	Ketamine 10 mg/kg i.p. or saline	PFC IL-1β, IL-6, and IL-10	Ketamine significantly decreased IL-1β (*p* < 0.01) and IL-6 (*p* < 0.05), and increased IL-10 (*p* < 0.05; anti-inflammatory)
				1 h	
Yang et al. (2020)	10 Adult male Sprague Dawley rats	Chronic postsurgical pain	Ketamine 20 mg/kg i.p. or saline	Hippocampal IL-1β, IL-6, and TNF-α	Ketamine significantly decreased IL-1β, IL-6 (*p* < 0.01), and TNF-α (*p* < 0.001). Only rats showing depression-like phenotype on behavioral tests were investigated
				7 days	
Zhang et al. (2016)	32 Adult male SD rats	Inflammatory pain-induced (CFA)	Ketamine 20 mg/kg i.p. or saline	Hippocampal IL-6, IL-1β, IDO, KYN, TRP, and 5-HT	Ketamine decreased IL-6 at 1 h and 24 h (*p* < 0.05), and IL-1β at 24 h (*p* < 0.01). IDO decreased at 1 h and 24 h (*p* < 0.01), KYN/TRP ratio decreased at 1 h and 24 h (*p* < 0.05), 5-HT/TRP ratio increased at 1 h and 24 h (*p* < 0.05)
				1 h or 24 h	
Zhao et al. (2020)	20 Male Wistar rats	LPS	Ketamine 10 mg/kg i.p. or no treatment	Hippocampal IL-1β, IL-6, TNF-α, and IDO	Ketamine decreased IL-6, TNF-α, and IDO (*p* < 0.01) and IL-1β (*p* < 0.05)
				2 h	
Zhu et al. (2015)	12 Adult male Sprague Dawley rats	CUMS	Ketamine 100 mg/kg i.p. + sham ECT or sham ECT only once daily for 7 days	hippocampal IL-1β and TNF-α (mRNA)	Ketamine decreased TNF-α (*p* < 0.05)
				9 days	

ACTH: adrenocorticotropic hormone; CFA: complete Freund’s adjuvant; CRP: C-reactive protein; CSF: cerebrospinal fluid; CUMS: chronic unpredictable mild stress; ECT: electroconvulsive therapy; FSL: Flinders sensitive line; FST: forced swim test; G-CSF: granulocyte colony-stimulating factor; GM-CSF: granulocyte-macrophage colony-stimulating factor; IDO: indoleamine 2,3-dioxygenase; IL: interleukin; IFN-α: interferon-alpha; i.p.: intraperitoneal; KYN: kynurenine; KynA: kynurenic acid; LPS: lipopolysaccharide; PFC: prefrontal cortex; QUIN: quinolinic acid; SNI: spared nerved injury; TNF-α: tumor necrosis factor-alpha; TRP: tryptophan.

a Numbers of rats indicate how many rats were included in the biomarker analysis in our groups of interest. If not mentioned otherwise, assumed that all rats in a group analyzed.

b Outcome measurement timepoints indicate the time from last ketamine administration (or LPS where this is after ketamine) to sample collection. If not mentioned otherwise, assumed that samples taken immediately after behavioral tests.

Quality scoring of animal studies are listed in Supplemental Table S2. Total quality scores ranged between four and eight out of a total of eight. Key differences in study quality arose from the depression model used, specifically whether induced depression was confirmed with behavioral testing ideally comparing performance to a group of nondepressed control rodents. There was also variability in clarity of study protocol and in comprehensiveness of reporting of outcome data and statistics for different comparisons.

### Results of individual studies and evidence synthesis

#### Human studies

Five out of six human studies measuring inflammatory proteins found decreases in at least one marker. Of these, one study stratified patients according to the responder status and found changes only in ketamine responders (Yang et al., 2015b). IL-1β decrease was observed in two out of three studies (*p* < 0.05; Yang et al., 2015b; Zhan et al., 2020); TNF-α in two out of five studies (*p* < 0.01; Chen et al., 2018; Zhan et al., 2020); IL-6 in three out of six studies (*p* < 0.01; Kiraly et al., 2017; Yang et al., 2015b; Zhan et al., 2020); and IFN-γ in one study out of three (*p* < 0.01; Zhan et al., 2020). Soluble tumor necrosis factor receptor 1 (sTNFR1) was measured in one study and was found reduced (*p* < 0.01; Park et al., 2017), likewise for IL-23 (Zhan et al., 2020), IL-1α, granulocyte colony-stimulating factor (G-CSF), platelet-derived growth factor (PDGF)-AA (*p* < 0.05), and interferon gamma-induced protein (IP)-10 (Kiraly et al., 2017). Additionally, IL-8 (*p* < 0.01), IL-13 (Kiraly et al., 2017), IL-2, IL-7, IL-10, IL-17A, fractalkine (*p* < 0.01; Zhan et al., 2020), granulocyte-macrophage colony-stimulating factor (GM-CSF), IL-4, IL-5, and IL-12p70 (*p* < 0.05; Zhan et al., 2020) were each decreased in one out of two studies. Finally, increases in cytokines IL-6 (Park et al., 2017) and IL-7 (Kiraly et al., 2017) were observed in one study each (*p* < 0.01).

Most changes in markers were short-term, lasting up to 240 min. However, Yang et al. (2015b) observed reductions sustaining for 1 and 3 days for IL-1β and IL-6, respectively, and Kiraly et al. (2017) observed reductions in IL-8 and PDGF-AA and increases in IL-7 at 24 h. Zhan et al. (2020) found no significant changes at the 24-h mark; however, levels of 14 proteins were reduced 2 week post-sixth ketamine infusion. In two of the five studies with significant findings, participants were maintained on their medications (Chen et al., 2018; Zhan et al., 2020), and in one study, BD subjects were allowed mood stabilizers (Park et al., 2017). In the study by Allen et al. (2018), who found no changes in any inflammatory protein, the majority of patients were medicated.

Changes in KYN metabolites indicating decreased inflammation were observed in two studies. Kadriu et al. (2019) found increased KYN, KynA, and KYN/KynA ratio and reduced IDO and QA/KYN ratio. These changes were long-term and were observed 1-day post-infusion for KYN/KynA and QA/KYN ratios, and 1- and 3-day post-infusion for KYN, KynA, and IDO; all *p*-values <0.01, except from KYN at day 3 (*p* < 0.05). Zhou et al. (2018) stratified patients according to the responder status and found changes only in ketamine responders; increased KynA and KynA/KYN ratio was measured at 24 h post-first and post-sixth ketamine infusion (all *p*-values <0.05), with the infusions given over a 12-day period. No changes were observed in TRP.

Yang et al. (2015b) and Moaddel et al. (2018) measured KYN and TRP but found no significant ketamine-induced changes over time. However, Moaddel et al. (2018) observed differential changes in markers when analyzed comparing to a placebo group, specifically a sharp initial drop in KYN and KYN/TRP ratio in the placebo group compared to slight increases with ketamine. Finally, Allen et al. (2018) measured KYN, KynA, and TRP with no significant results, but observed a trend toward decreased KYN at 2 h (*p* = 0.067) and KYN/TRP ratio at 24 h (*p* = 0.054) in responders.

#### Animal studies

About 18 out of 21 animal studies found reductions in one or more pro-inflammatory markers. Decreased IL-1β was observed in 14 out of 19 studies and decreased IL-6 in 13 out of 18 studies. Decreased TNF-α was found in nine out of 14 studies; additionally, a study on adrenocorticotropic hormone (ACTH)-induced treatment resistance by Walker et al. (2015) observed decreased TNF-α in ketamine nonresponders compared to a placebo group, *p* < 0.05. One study recorded an unexpected surge in TNF-α (Abelaira et al., 2017). IL-1α was measured in one study and found decreased, *p* < 0.01 (Verdonk et al., 2019). One study out of five also observed an increase in anti-inflammatory cytokine IL-10 (*p* < 0.05; Yang et al., 2013b). Walker et al. (2015) observed elevated CRP in ACTH-treated ketamine responders compared with placebo group, *p* < 0.05. IFN-γ was measured twice with no differences found (Clarke et al., 2017; Verdonk et al., 2019).

Four out of six studies measuring TRP metabolites found changes indicating decreased inflammation. Verdonk et al. (2019) observed increased KynA and decreased QUIN and 3-HK in ketamine-treated mice (*p* < 0.01); however, no changes in these three were observed by Eskelund et al. (2017). KYN/TRP ratio was decreased in two studies out of five (*p* < 0.05; Wang et al., 2015; Zhang et al., 2016), but no differences were found in absolute levels of KYN or TRP. IDO was decreased in three studies out of four, all *p*-values <0.01 (Wang et al., 2015; Zhang et al., 2016; Zhao et al., 2020). Two studies found no significant differences in any metabolite of the pathway (Eskelund et al., 2017; Walker et al., 2013).

No remarkable differences appeared between results of studies measuring central versus peripheral markers. Of studies measuring both, Reus et al. (2015) found ketamine to reduce IL-6 in both serum and CSF, but IL-1β and TNF-α in serum only (all *p*-values <0.05). One study found overall levels of plasma and brain KYN metabolites to be strongly correlated, though main analyses of ketamine’s effect were only done in brain markers (Verdonk et al., 2019). No marked differences appeared between samples obtained at different timepoints.

There was an indication for dose-dependency, with higher doses showing more robust effects. Three out of four studies with a low ketamine dose of 5 or 6 mg/kg found no significant results, with one observing a decrease only in TNF-α but not in IL-1β, which was decreased with a higher 10 mg/kg infusion in the same study (Clarke et al., 2017). One study found reductions in three pro-inflammatory markers after both 10 and 90 mg/kg doses; however, all changes were larger following the higher dose (Verdonk et al., 2019).

## Discussion

This study is the first systematic review examining the effect of ketamine on both inflammation and TRP–KYN pathway in depression in clinical as well as preclinical animal studies. Preclinical evidence has brought strong evidence for ketamine’s anti-inflammatory and neuroprotective properties, with nearly all null findings occurring in studies where a low-dose ketamine was used. However, studies in humans are thus far scarce and have yielded more mixed findings, they overall seem to support decreases in inflammation and activation of neuroprotective branch of the KYN pathway, at least in a subset of patients.

Ketamine-induced reductions of inflammatory markers were observed most commonly for the cytokines IL-1β, IL-6, and TNF-α, which have all been consistently found to be implicated in depressive illness (Borsini et al., 2020; Osimo et al., 2020; Raison et al., 2006; Rosenblat et al., 2014). These three cytokines are known to upregulate the enzyme IDO, which converts TRP into KYN (Maes et al., 2007); additionally, IL-1β enhances expression of KMO that further converts KYN into its neurotoxic metabolites (Moffett and Namboodiri, 2003). Besides effect on TRP metabolism, pro-inflammatory cytokines contribute to depressive symptoms by disrupting monoamine metabolism and hypothalamic–pituitary–adrenal (HPA) axis function (Felger and Lotrich, 2013; Nikkheslat et al., 2018, 2020) and also by directly influencing glutamate signaling contributing to glutamate excitotoxicity and reduced BDNF (Haroon and Miller, 2016; Miller et al., 2009). It is notable that in many clinical studies changes in inflammatory proteins did not sustain past 24 h, and it is uncertain whether such transient changes can inflict cascades of downstream events that are implicated in ketamine’s long-term antidepressant effects. Observations of KYN metabolite changes of up to 3 days support this possibility, but further research is called for. The ketamine response has previously been found to be associated with increased BDNF up to 1-week post-infusion (Allen et al., 2015; Haile et al., 2014), yet it is unknown to what extent this is induced by ketamine’s anti-inflammatory effects in contrast to the drug’s direct effect on glutamatergic signaling or other mechanisms.

The IDO was found decreased in all but one study it was measured, supporting ketamine’s anti-inflammatory action through decreasing pro-inflammatory cytokines and subsequently downregulating the activity of the enzyme. In animal models, IDO activity has been found essential for inflammation-induced depressive symptoms (Lawson et al., 2013; O’Connor et al., 2009). In contrast, a large longitudinal study in depressed patients found that KYN/TRP ratio, a commonly used indirect indicator of IDO activity, did not mediate the relationship between inflammation and depressive symptoms; in fact, KYN/TRP ratio was even lower in depressed patients though this was no longer significant after adjusting for antidepressant use (Quak et al., 2014). However, validity of KYN/TRP ratio as a proxy for IDO has been challenged (Badawy and Guillemin, 2019), which may also help explain why reductions in this ratio were observed only in two studies of this review despite more consistently observed changes in IDO. In future investigations, direct measurement of IDO is endorsed for reliable evidence of the enzyme’s activity.

Although decreased IDO may lead to reduced synthesis of KYN, our review found no consistent evidence for changes in this metabolite. Changes in KYN may be less detectable due to its eventual transamination into downstream metabolites (Badawy and Guillemin, 2019), levels of which are arguably more relevant markers for ketamine’s action and anti-inflammatory effects. Indeed, as an indicator of inflammation, increased IDO only leads to neurotoxicity with additional upregulation of KMO and subsequent conversion of KYN into its neurotoxic metabolites. Our review found evidence for increased KynA and reduced QUIN following ketamine in both clinical and preclinical studies, alongside decreased 3-HK in one animal study, supporting anti-inflammatory activity and activation of KAT over KMO and a shift toward neuroprotective rather than neurotoxic pathway. The effect of ketamine in decreasing pro-inflammatory cytokines leads to less activity of KMO and increased availability and synthesis of KYN into KynA instead of QUIN. KMO has been found essential for inflammation-induced depression in rodents (Parrott et al., 2016), suggesting the present results could be of high clinical relevance. However, due to the small number of studies measuring these metabolites as presented in our review, further research is needed.

Heterogeneity in inclusion criteria and methodologies of included human studies not only complicate the interpretation of the evidence base but also shed light on the potential reasons for the inconsistencies seen in the literature. Studies without significant findings appeared either underpowered and were on medicated patients (Allen et al., 2018) or measured only a few markers and applied less strict treatment-resistant criteria (Moaddel et al., 2018). Given inflammation levels are generally lower in nontreatment-resistant patients (Cattaneo et al., 2020; Strawbridge et al., 2015; Yang et al., 2019),; and traditional antidepressants (Köhler et al., 2018; Strawbridge et al., 2015) and mood stabilizers (Li et al., 2015; Rapaport et al., 1999) have been found to exert anti-inflammatory effects, ketamine might be less likely to show anti-inflammatory effects in nontreatment-resistant and in medicated subjects. It is yet worth noting that even in many higher-quality studies decreases were observed only in one or two inflammatory markers. The two studies that stratified results according to the responder status to antidepressants only found significant results in those who responded, supporting the possibility that ketamine’s antidepressant effect might at least partially be mediated through its anti-inflammatory effects, which are more prominent in responders (Yang et al., 2015b; Zhou et al., 2018).

Of relevance, surgical and preclinical research has indicated ketamine does not reduce inflammation unless it is abnormally high (Loix et al., 2011); whether and to what extent baseline inflammation predicts ketamine’s effect on inflammatory markers as well as symptomatic improvement of depression are important areas for further investigation. In the study by Yang et al. (2015b), who found both higher baseline levels of, as well as ketamine-induced reductions in, IL-6 and IL-1β to be associated with response status, baseline levels of these markers were remarkably higher than seen in most populations. Variability in baseline inflammation levels within and between studies could also be one reason why preclinical findings have not been replicated as consistently in humans; in rodents, depression is induced mechanistically and in many times with inflammatory stimuli, providing optimal condition for ketamine to demonstrate its anti-pro-inflammatory effects. The only animal study with a moderate-to-high ketamine dose but with no significant findings used a genetic rat model of depression (Eskelund et al., 2017), which had previously demonstrated *lower* levels of QUIN and no difference in KYN/TRP levels compared to its selectively bred controls (Eskelund et al., 2016). It is also notable that in several rodent studies ketamine was administered *before* inflammatory challenge, which can result in more potent anti-inflammatory effects than when administered once inflammation is already present (Taniguchi et al., 2001).

There was a strong indication for dose-dependency across animal studies, and likewise one clinical trial found anti-inflammatory effects only following 0.5 mg/kg but not 0.2 mg/kg ketamine infusion (Chen et al., 2018). Potential increased efficacy of even higher ketamine doses in humans is unclear. In surgical contexts, subanesthetic doses similar or even lower to antidepressant doses have been found sufficient for inducing anti-inflammatory effects (Dale et al., 2012). Evidence of antidepressant efficacy of higher ketamine doses is mixed with one study showing no superiority of 1.0 mg/kg over 0.5 mg/kg infusion (Fava et al., 2018), but another indicating more pronounced responses after doses were escalated from 0.5 mg/kg to 0.75 mg/kg (Cusin et al., 2017). Hypothetically, it remains plausible higher ketamine dose could also trigger or augment anti-inflammatory effect in some patients in whom this was not evident and could therefore be investigated in the future. Research on the effect of multiple ketamine infusions is also crucial, given repeated dosing is commonly needed and used in practice (Phillips et al., 2019; Voort et al., 2016).

It is important to highlight that the included human studies only measured circulating markers, which might not provide a reflection of levels in the central nervous system. Regarding TRP metabolites, KYN, TRP, and 3-HK readily cross the blood–brain barrier, but QUIN and KynA do this at lower rates and are instead produced in the brain immune cells (Guillemin, 2012; Schwarcz and Pellicciari, 2002). Although inflammation has previously been suggested to increase blood–brain barrier permeability (Skaper, 2017), this notion has recently been challenged (Turkheimer et al., 2020) and robust consistent associations between brain and blood inflammation, or KYNs are yet to be found in depression (Nettis and Pariante, 2020). A recent study in unmedicated-depressed patients showed that while plasma and CSF levels of KYN, KYN/TRP ratio, and QUIN correlated strongly, there was no significant relationship for KynA, TRP, and QUIN/KynA ratio (Haroon et al., 2020). In the same study, a significant relationship was found between plasma and CSF CRP and IL-6 soluble receptor, but correlations were close to zero for most other inflammatory proteins including TNF, IL-6, and IL-1β (Haroon et al., 2020). Studies examining markers in the CSF or in the brain with positron emission tomography are therefore urgently needed.

There are some limitations with the current review that may help for directions of future studies. The number of clinical studies on the effect of ketamine is still small, and relying only on using animals to model complex psychiatric conditions and investigate the effectiveness of drugs would not be an ideal approach (O’Leary and Cryan, 2013). Gray literature was not searched in this review. Meta-analysis could not be conducted due to variability between methodologies and biomarkers measured of the included studies. Heterogeneity between studies, namely in diagnoses, disease profile, potential comorbidities, medication status, and analysis methods, highlights the need for research with more comparable inclusion criteria and methodology. Further, IFN-γ was only measured three times and CRP twice; IFN-γ is the key cytokine activating IDO (Maes et al., 2007); and CRP has been found as a predictor for response to antidepressants with anti-inflammatory characteristics (Yang et al., 2019); therefore, inclusion of these markers in future research would be of interest. Future studies should also ideally include a placebo group, highlighted by saline-induced fluctuations in markers observed in some studies (Chen et al., 2018; Moaddel et al., 2018).

## Conclusion

In conclusion, the present review supports ketamine’s anti-inflammatory effects in depressed humans and rodents. Ketamine is considered the most effective antidepressant available for treatment-resistant patients and for suicide prevention, enhancing understanding of its pharmacology is crucial for the development of precision medicine, understanding of neurobiological mechanisms underlying depression, and identification of therapeutic targets for other novel antidepressants with potentially better side effect profile and less abuse liability. Crucial next steps for further research include investigation of the specific molecular mechanism behind ketamine’s immunomodulatory effects, examination of clinical relevance of inflammatory changes, and measurement of markers in the central nervous system.

## Supplemental Material

sj-docx-1-jop-10.1177_02698811211026426 – Supplemental material for Ketamine’s effect on inflammation and kynurenine pathway in depression: A systematic review Click here for additional data file. Supplemental material, sj-docx-1-jop-10.1177_02698811211026426 for Ketamine’s effect on inflammation and kynurenine pathway in depression: A systematic review by Emma Kopra, Valeria Mondelli, Carmine Pariante and Naghmeh Nikkheslat in Journal of Psychopharmacology
