# Supplementary material for: Ketamine’s effect on inflammation and kynurenine pathway in depression: A systematic review
Source: J Psychopharmacol. 2021 Jun 26;35(8):934–45. doi: 10.1177/02698811211026426 (PMC8358579; doi:10.1177/02698811211026426)
Supplement: sj-docx-1-jop-10.1177_02698811211026426 – Supplemental material for Ketamine’s effect on inflammation and kynurenine pathway in depression: A systematic review [file sj-docx-1-jop-10.1177_02698811211026426.docx]

## Supplementary Material

**Figure S1**

*
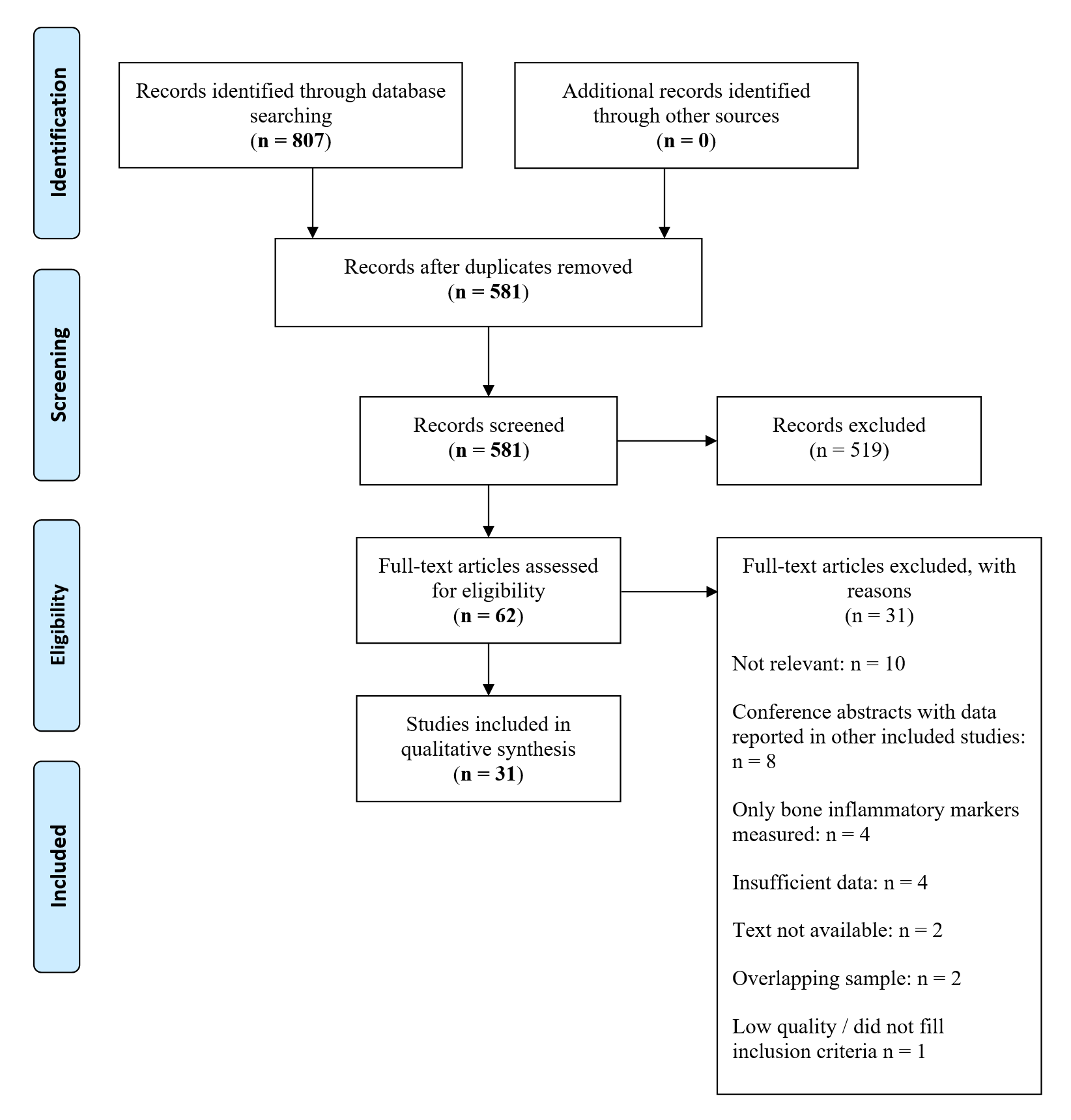
Prisma flow diagram.*

**Table S1**

| Study | Blinding | | Free of medication | | Measurement freq | | Statistical analyses | | Data and reporting | | Total |  |
| --- | --- | --- | --- | --- | --- | --- | --- | --- | --- | --- | --- | --- |
| Allen et al. | 0 | 0 | | 2 | | 1 | | 1 | | 4 | | |
| Chen et al. | 2 | 0 | | 2 | | 2 | | 1 | | 7 | | |
| Kadriu et al. | 2 | 1 | | 1 | | 1 | | 2 | | 7 | | |
| Kiraly et al. | 0 | 2 | | 1 | | 1 | | 2 | | 6 | | |
| Moaddel et al. | 2 | 2 | | 2 | | 0 | | 2 | | 8 | | |
| Park et al. | 2 | 1 | | 1 | | 2 | | 2 | | 8 | | |
| Yang et al. | 0 | 2 | | 1 | | 1 | | 2 | | 6 | | |
| Zhan et al. | 0 | 0 | | 1 | | 2 | | 2 | | 5 | | |
| Zhou et al. | 0 | 0 | | 1 | | 2 | | 2 | | 5 | | |

*Quality scoring of human studies.*

**Table S2**

*Quality scoring of animal studies.*

| Study | Depression model | Protocol | Statistical analyses | Data and reporting | Total |
| --- | --- | --- | --- | --- | --- |
|  |  |  |  |  |  |
| Abelaira et al. | 1 | 2 | 2 | 2 | 7 |
| Aricioglu et al. | 2 | 2 | 2 | 1 | 7 |
| Chang et al. | 2 | 2 | 2 | 1 | 7 |
| Clarke et al. | 2 | 2 | 2 | 0 | 6 |
| Eskelund et al. | 1 | 2 | 2 | 2 | 7 |
| Ji et al. | 2 | 2 | 2 | 1 | 7 |
| Li et al. | 2 | 1 | 2 | 1 | 6 |
| Reus et al. 2017 | 0 | 1 | 2 | 1 | 4 |
| Reus et al. 2015 | 2 | 2 | 2 | 2 | 8 |
| Tan et al. | 2 | 2 | 2 | 1 | 7 |
| Unal et al. | 1 | 1 | 2 | 0 | 4 |
| Verdonk et al. | 2 | 2 | 2 | 1 | 7 |
| Walker et al. 2015 | 1 | 2 | 2 | 2 | 7 |
| Walker et al. 2013 | 2 | 2 | 2 | 2 | 8 |
| Wang et al. | 2 | 2 | 2 | 1 | 7 |
| Xie et al. | 2 | 2 | 2 | 1 | 7 |
| Yang et al. 2013b | 1 | 2 | 2 | 1 | 6 |
| Yang et al. 2013a | 2 | 2 | 2 | 2 | 8 |
| Yang et al. 2020 | 2 | 2 | 2 | 2 | 8 |
| Zhang et al. | 2 | 2 | 2 | 1 | 7 |
| Zhao et al. | 2 | 1 | 2 | 1 | 6 |
| Zhu et al. | 2 | 1 | 2 | 2 | 7 |
